# *Acanthus mollis* Leaf Extract as Potential New Food Ingredient in the Prevention of Aging-Related Neurodegeneration

**DOI:** 10.3390/foods15111907

**Published:** 2026-05-28

**Authors:** Valeria Cavalloro, Giulia Moretto, Alice Fossati, Francesco Saverio Robustelli della Cuna, Simona Collina, Emanuela Martino, Raffaella Colombo, Adele Papetti

**Affiliations:** 1 Department of Earth and Environmental Sciences, University of Pavia, Via Ferrata 1, 27100 Pavia, Italy; valeria.cavalloro@unipv.it (V.C.); alice.fossati01@universitadipavia.it (A.F.); 2 Department of Drug Sciences, University of Pavia, Viale Taramelli 12, 27100 Pavia, Italy; giulia.moretto01@universitadipavia.it (G.M.); simona.collina@unipv.it (S.C.); raffaella.colombo@unipv.it (R.C.); adele.papetti@unipv.it (A.P.); 3 NBFC—National Biodiversity Future Center, Piazza Marina 61, 90133 Palermo, Italy; 4 Environmental Research Center, ICS Maugeri SPA SB, Institute of Pavia, IRCCS, Via Maugeri 2, 27100 Pavia, Italy; fsaveriorobustelli@unipv.it

**Keywords:** DIBOA, verbascoside, AGE, aging-related disorders, tyrosinase, nature-aided drug discovery

## Abstract

Life expectancy in high-income countries is increasing, leading to a higher incidence of age-related neurodegenerative diseases. To address this urgent medical need, several molecular targets have been identified, including advanced glycation end products (AGEs) and tyrosinase. Given the well-established role of diet in counteracting degenerative processes, this study aimed to identify a potential food ingredient with combined anti-tyrosinase and anti-glycative properties. *Acanthus mollis* L. was selected based on its inclusion in the BelFrIt list and its known content of tyrosinase inhibitors, such as benzoxazinones and verbascoside. Extraction of *A. mollis* leaves was optimized using a design of experiments approach, comparing microwave- and ultrasound-assisted techniques. Optimal conditions were achieved using microwave-assisted extraction with ethanol 80%, 80 °C, one cycle, drug-to-solvent ratio of 10 mL/g. The optimized extract (at 5 mg/mL) inhibited tyrosinase activity by approximately 47%, increasing to 58% after chlorophyll removal. Moreover, the extract reduces AGEs formation in presence of methylglyoxal, with an activity at 1 mg/mL comparable with that of a well-known anti-glycative agent. A similar trend was observed in the reduction in methylglyoxal and glyoxal levels. Overall, these results support the potential of the optimized *A. mollis* extract as a functional food ingredient to counteract aging-related neurodegeneration.

## 1. Introduction

Over the last century, the lifespan of high-income countries has significantly risen, owing mainly to improved hygienic conditions and available medical practices [1]. However, the increasingly older population brings to the table new challenges for public health management, mainly due to the associated increase in chronic degenerative diseases. These pathologies group a wide range of diseases, like metabolomic syndromes, chronic inflammations, cardiovascular or neurodegenerative diseases, and cancer, and they have been the main causes of disability and death worldwide. Given the estimated negative trend of chronic degenerative diseases incidence, finding new preventive strategies should be considered of pivotal importance for current research [2]. Diet plays a crucial role in this field. Thus, it has already been demonstrated that the Mediterranean diet can prevent many chronic degenerative diseases, owing to both its balanced nutritional content and the biological activities of the secondary metabolites found in foods [3]. Furthermore, many functional foods have been studied to improve brain health and prevent aging. The success of these products is mainly linked to the possibility of exploiting the entourage effect, which is particularly important in multifactorial pathologies. Therefore, the different components of food ingredients may interact with different pathways, synergistically cooperating to prevent chronic degenerative diseases. Particularly, many pathways could be targeted, like the one associated with advanced glycation end products (AGEs). AGEs are a heterogeneous and complex group of compounds generated via the Maillard reaction, a series of non-enzymatic browning reactions between the carbonyl groups of reducing sugars and the free amino groups of proteins, followed by further rearrangements that lead to the formation of dangerous carbonyl intermediates, specifically methylglyoxal (MGO) and glyoxal (GO), until the development of stable and irreversible end products, which are considered the most toxic species [4]. Foods rich in proteins, sugars and oxidized lipids together with processed food (baked, roasted, and fried) at high temperatures are potential sources of AGEs [5,6]. Also, the choice of certain cooking methods/conditions (boiling, steaming poaching) and addition of specific ingredients (acidic agents or polyphenols) can provide important strategies to reduce AGE formation [7].

**Figure 1:**
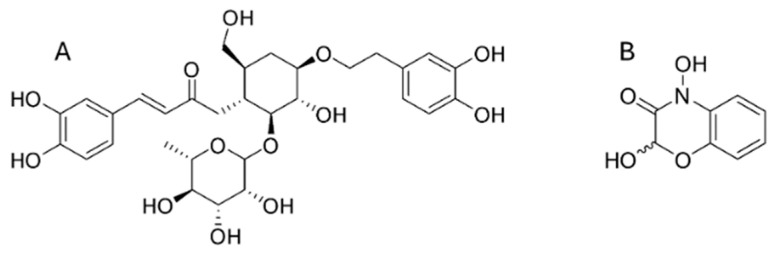
Chemical structure of verbascoside (**A**) and DIBOA (**B**).

The glycation reaction is particularly promoted by hyperglycemia, high-fat diet and oxidative stress, and the accumulation of AGEs in the body is associated with the development and progression of several chronic degenerative disorders, such as diabetes and cardiovascular and neurodegenerative diseases [8]. In fact, AGEs can modify the proteins’ structure, forming crosslinks within the same protein or between different proteins, making them more rigid and less functional [9]. In addition, they can bind the receptor for advanced glycation end products (RAGEs), leading to the activation of inflammatory signaling pathways and increased oxidative stress, causing cell damage [10]. However, secondary metabolites demonstrated promising anti-glycative properties, as they can reduce AGE formation by protecting protein glycation sites, reducing oxidative stress, trapping dicarbonyl compounds or modulating RAGEs [11]. Therefore, identifying new food ingredients able to interfere with AGE formation may represent a new step ahead in the fight against chronic degenerative diseases [12,13]. To this aim, in the present study we focus on *Acanthus mollis* L., being part of the BelFrIt list. This project derives from the joint effort of Belgium, France, and Italy, which allowed for editing a list of more than one hundred plants (or their parts) that, based on the current knowledge, can be considered potential ingredients of food supplements. Despite it being native to the Mediterranean regions, *A. mollis* has been introduced in many territories with temperate, subtropical, and tropical climates due to its popularity as an ornamental plant [14,15]. Its adaptability and diffusion are particularly important, allowing easy access to biomass for researchers and industries. Another important characteristic that makes *A. mollis* a suitable candidate as a source of food ingredient is its metabolomic content. Thus, its ethanolic extract has already demonstrated cytocompatibility with different cell lines [16]. Furthermore, in its extract two main metabolites were identified: verbascoside, also known as kusaginin, and the benzoxazinone derivative 2,4-dihydroxy-1,4-benzoxazin-3-one (DIBOA) (Figure 1) [17].

Both these metabolites showed a promising anti-tyrosinase activity, with IC_50_ values of 27 μM and 23 μM, respectively [16]. Tyrosinase, primarily known for its major role in melanogenesis in the skin, has also been found in the human brain, specifically in *substantia nigra* dopaminergic neurons. There, it catalyzes the conversion of L-tyrosine to L-DOPA, which is further oxidized into neuromelanin, a dark brown pigment, with the production of ROS and toxic metabolites [18]. Neuromelanin accumulation in dopaminergic neurons makes them particularly susceptible to neurodegeneration, making tyrosinase a promising target to contrast these degenerative pathologies.

Furthermore, verbascoside is endowed with anti-inflammatory, anti-glycative [19,20], and antioxidant activities [21] and it inhibits monoamine oxidase A (MAO-A) in a dose-dependent manner (IC_50_ = 3 μM in SH-SY5Y cells) [22]. MAO isoforms play a crucial role in the catabolism of monoaminergic neurotransmitters, and its inhibition leads to an increase in levels of serotonin and noradrenalin. Taken together, all these activities make the extract obtained from *A. mollis* an interesting source of new ingredients to counteract chronic degenerative diseases, particularly those linked with neurodegeneration.

Starting with this evidence, the present work aims to optimize a scaling-up extraction procedure, combining microwave-assisted extraction (MAE) and ultrasound-assisted extraction (UAE), characterize the main metabolites by the RP-UHPLC-DAD method, and investigate anti-tyrosinase and anti-glycative activity of *A. mollis* leaves extract. Due to the already investigated activities of its main metabolites, the main future focus here is the development of a suitable and effective oral formulation of *A. mollis* leaves extract that can potentially prevent aging-associated pathological conditions.

## 2. Materials and Methods

### 2.1. Reagents

HPLC-grade acetic acid, formic acid, and methanol, deuterated methanol, methylglyoxal (MGO, 40% aqueous solution), glyoxal (GO, 40% aqueous solution), 5-methylquinoxaline (5-MQ, purity grade ≥ 98%), o-phenylenediamine (OPD, purity grade ≥ 98%), aminoguanidine hydrochloride (AG, purity grade ≥ 98%), bovine serum albumin (BSA, purity grade ≥ 98%), sodium dihydrogen phosphate monohydrate (purity grade ≥ 98%), disodium hydrogen phosphate dodecahydrate (purity grade ≥ 99%), sodium azide (purity grade ≥ 99.5%), tyrosinase (enzyme and subtrates) were provided by Merck Life Science S.r.l. (Milan, Italy). Water was obtained from a Millipore Direct-QTM system (Merck-Millipore, Milan, Italy). Verbascoside standard was purchased by Extrasynthese (Genay, France).

### 2.2. Plant Material

Leaves of *Acanthus mollis* were harvested from the botanical garden of the University of Pavia (coordinates: 45°11′08.99″ N 9°09′47.27″ E). The samples were dried at 35 °C in a ventilated oven and then stored in a dry place away from direct light.

### 2.3. Isolation and Identification of Main Metabolites

A preliminary extract was prepared by applying a MAE protocol previously developed in-house on 5 g of *A. mollis* powdered leaves using ethanol as a solvent (2 min ramping, maximum pressure 120 psi, maximum power 100 W, 50 °C, Discover® Lab-Mate instrument, CEM Corporate, Buckingham, UK) for three cycles of 5 min each. The extract was then filtered and the solvent evaporated under reduced pressure. The extract obtained was solubilized in methanol (final concentration 10 mg/mL), filtered through a 0.45 μm GH Polypro membrane (GHP-PerkinElmer, Shanghai, China), and fractionated via (semi)-preparative HPLC (Jasco, Tokyo, Japan). The applied method involved the use of a Chromolith SemiPrep RP-18 endcapped 100 × 10 mm column (Merck, Darmstadt, Germany), flow rate of 4 mL/min, and 100 μL of injection volume. Analyses were performed in gradient conditions using water as solvent A and methanol as solvent B, following the subsequent gradient conditions: from 95% to 50% A in 10 min, 1 min 5% A kept constant for 2 min, followed by a re-equilibration step of 4 min.

Verbascoside, [M-H]^−^ *m*/*z* 623; ^1^H NMR (400 MHz, MeOD) δ 7.59 (d, J = 15.9 Hz, 1H), 7.06 (d, J = 2.1 Hz, 1H), 6.96 (dd, J = 8.2, 2.1 Hz, 1H), 6.78 (d, J = 8.1 Hz, 1H), 6.70 (d, J = 2.1 Hz, 1H), 6.68 (d, J = 8.0 Hz, 1H), 6.57 (dd, J = 8.1, 2.1 Hz, 1H), 6.27 (d, J = 15.9 Hz, 1H), 5.19 (d, J = 1.8 Hz, 1H), 4.38 (d, J = 7.9 Hz, 1H), 4.05 (ddd, J = 9.7, 8.0, 6.8 Hz, 1H), 3.92 (dd, J = 3.3, 1.8 Hz, 1H), 3.82 (t, J = 9.2 Hz, 1H), 3.73 (ddd, J = 9.7, 8.1, 6.8 Hz, 1H), 3.66–3.50 (m, 4H), 3.43–3.34 (m, 1H), 3.27 (s, 0H), 2.80 (ddd, J = 8.4, 6.9, 2.3 Hz, 2H), 1.09 (d, J = 6.2 Hz, 3H).

DIBOA-glc, [M+HCOO]^−^ *m*/*z* 388; ^1^H NMR (400 MHz, MeOD) δ 7.33 (d, J = 7.6 Hz, 1H), 7.05–6.93 (m, 3H), 6.95–6.88 (m, 0H), 5.81 (s, 1H), 5.65 (s, 0H), 5.59 (s, 0H), 4.57 (d, J = 7.9 Hz, 1H), 3.75 (dd, J = 11.9, 1.7 Hz, 1H), 3.63–3.53 (m, 1H), 3.25 (s, 1H), 3.24–3.17 (m, 5H), 3.13–3.01 (m, 1H).

### 2.4. Extraction Procedure

Two methods, MAE and UAE (Elmasonic P 120 H, Elma Schmidbauer GmbH, Singen, Germany), were employed. Particularly, MAE extraction was carried out using a power of 70 W, a maximum pressure of 120 psi, a ramp time of 2 min, and an extraction time of 5 min for each cycle under magnetic stirring. Further, UAE extraction was carried out using a working frequency of 80 kHz, an electric power output of 450 W, and an extraction time of 10 min for each cycle. All these parameters were selected based on our previous experience [23]. Other factors were studied by applying a full factorial design to simultaneously compare their effect on the extraction yield of verbascoside (Y_1_) and DIBOA-glc (Y_2_). Both responses were quantified by HPLC analysis using the external calibration curve method (see Section 2.5).

The experimental plan consisted of four continuous factors varying over two levels. Factor X_1_ was the solvent-to-solid ratio (from 10 to 30 mL/mg), X_2_ was the temperature (from 40 to 80 °C), X_3_ was the number of extraction cycles (from 1 to 3 cycles), and X_4_ was the solvent composition (20% to 80% ethanol in water).

Three additional independent experiments were added to the experimental plan to test the model’s validity (experiments 17, 18, and 19). The design of experiment plan is described in Table 1.

After each extraction, the mixture was allowed to cool to room temperature and filtered using vacuum filtration with a Büchner funnel. All samples were dried to constant weight.

### 2.5. RP-UHPLC-DAD Analysis of Acanthus mollis Extract and Its Main Metabolites

A suitable RP-UHPLC-DAD method was set up for the qualitative and quantitative analysis of the crude extract resulting from MAE and UAE procedures (as detailed above) and its main metabolites, including verbascoside and DIBOA-glc isolated as described in Section 2.3. Analyses were performed using a Vanquish UHPLC system (Thermo Fisher Scientific, Waltham, MA, USA) equipped with an autosampler, binary pump, column compartment, and diode array detector (DAD). Chromatographic separation was carried out on a Gemini^®^ NX-C18 analytical column (150 × 2.0 mm i.d., 3 μm, Phenomenex, Torrance, CA, USA). The flow rate was set at 0.3 mL/min and an injection volume of 10 µL was applied. The mobile phase consists of 0.1% formic acid aqueous solution (A) and methanol (B) with the following gradient: 0 min 5% B, 3.75 min 15% B, 5.55 min 25% B, 14.55 min 50% B, 18.15 min 80%, 20 min 95%, 22 min 5% B, 32 min 5% B. The column temperature was set at 25 °C, and chromatograms were recorded at 280 and 320 nm, corresponding to the maximum absorbance of DIBOA-glc and verbascoside, respectively.

The method validation was performed according to the International Conference on Harmonization (ICH) guidelines Q2(R2) (2022), considering linearity, accuracy, precision, limit of detection (LOD), and limit of quantification (LOQ) [24]. A calibration curve for verbascoside and DIBOA-glc was prepared at seven concentration levels (1–150 µg/mL and 5–150 µg/mL, respectively) with each level analyzed in triplicate to evaluate linearity using the external standard method. The area of verbascoside (Y_1_) and DIBOA-glc (Y_2_) registered at 320 and 280 nm, respectively, was plotted against the corresponding theoretical concentrations to obtain the calibration curve, and linear regression analysis was performed using the least-squares method. A correlation coefficient (R^2^) ≥ 0.9900 was considered acceptable. LOD and LOQ were calculated based on signal-to-noise (S/N) ratios of approximately 3 and 10, respectively. Precision was assessed by analyzing each concentration level in triplicate, evaluating both intra-day and inter-day (across three days) precision, and expressed as relative standard deviation (RSD%). Accuracy was evaluated at three concentration levels (1, 50, and 150 µg/mL and 5, 50 and 150 µg/mL for verbascoside and DIBOA-glc, respectively), corresponding to low, medium, and high concentrations. For each level, samples were analyzed in triplicate, and recovery was calculated as the percentage ratio between the measured and theoretical concentrations of compound.

Verbascoside and DIBOA-glc stock solutions were prepared by dissolving the pure compounds in methanol, followed by dilution with 0.1% formic acid aqueous solution and methanol 50:50 *v*/*v* to obtain each concentration level.

Dried crude extracts were dissolved in a proper volume of 0.1% formic acid aqueous solution and methanol 50:50 *v*/*v* to obtain a final solution of 0.5 mg/mL.

### 2.6. Extraction Scale-Up

The best extraction conditions were scaled up using 5 g of starting biomass. The instrument was set as follows: power of 320 W, maximum pressure of 120 psi, matrix-to-solvent ratio of 10 mL/mg, ramp time of 2 min, extraction time of 5 min, 1 cycle, 80 °C, and 80% ethanol in water as solvent (MARSX system, CEM Corporate, Buckingham, UK). The mixture was allowed to cool to room temperature, filtered through vacuum filtration apparatus, and finally the solvents were evaporated under reduced pressure (rotary evaporator followed by smart evaporator).

### 2.7. Chlorophyll Removal

Before biological investigation, chlorophylls were removed from an aliquot of the extract obtained after the scale-up. A solid phase extraction (SPE) method recently developed by us was applied [25]. Briefly, the cartridges Waters OASIS HLB 1 cc (Waters, Milford, MA, USA) were mounted on the manifold and conditioned with 1 mL of methanol. An amount of 1 mL of each extract previously dissolved in methanol (c = 5 mg/mL) was loaded into cartridges; the vacuum was applied and the cartridge washed with 1 mL methanol. At the end, the solvent was evaporated under reduced pressure to obtain the purified extracts.

### 2.8. Tyrosinase Inhibition Assay

In brief, the activity of mushroom tyrosinase (in phosphate buffer 50 mM pH 6.8 solution at 1000 U/mL) was monitored colorimetrically using L-tyrosine as a substrate (at 0.5 mg/mL in distilled water) and a sample solution as an inhibitor (5 mg/mL in methanol) for dopachrome formation [26,27]. The reaction mix of the test well was composed of 60 μL of L-tyrosine solution, 15 μL of enzyme solution, 30 μL of extract, and 195 μL of phosphate buffer to reach a total of 300 μL per well. The reaction mix of the control well was composed of 60 µL of L-tyrosine, 15 µL of tyrosinase, and 225 µL of phosphate buffer, and the reaction mix of the blank well was composed of 30 µL of extract solution and 270 µL of phosphate buffer. Arbutin was used as the positive control at different concentrations (200 μg/mL, 100 μg/mL, 50 μg/mL, 25 μg/mL, 125 μg/mL, and 62.5 μg/mL in distilled water) and seeded following the test well composition of the reaction mix. The plate was left to incubate in the dark at room temperature for 30 min; later the absorbance was measured at 480 nm on a microplate reader. The inhibition activity of the extracts is calculated by applying the following equation:

$$Inhibition(\%)=[1-\frac{\left(Abs\;sample-Abs\;blank\right)}{Abs\;control}\;]\;\times \;100$$

All tests were performed in triplicate.

### 2.9. Evaluation of the Extract’s Trapping Activity by the RP-UHPLC-DAD Method

The capacity of optimized *A. mollis* extract (before and after chlorophyll removal) to trap glyoxal (GO) or methylglyoxal (MGO) was evaluated following the method described by Maietta et al., 2018, with slight modifications [28]. Extract solutions were prepared by dissolving the dried material in water, followed by dilution with phosphate buffer (100 mM, pH 7.4, containing 0.02% NaN_3_) to obtain a final concentration of 0.25, 0.5, and 1 mg/mL in the reaction mixture. Then, MGO or GO was incubated for 24, 48, 72, and 96 h at 37 °C with or without the extract solution. The amount of trapped GO and MGO was monitored by derivatization of the residual GO and MGO into their corresponding quinoxaline derivatives, followed by RP-UHPLC-DAD analysis, as described by Moretto et al., 2025 [20]. The trapping capacity of the optimized *A. mollis* extract was calculated as follows and expressed as the percentages of trapped MGO or GO:

$$\mathrm{GO}\;\mathrm{or}\;\mathrm{MGO}\;\mathrm{trapped}\;(\%)\;=\left[\frac{(\;\mathrm{GO}\;\mathrm{or}\;\mathrm{MGO}\;\mathrm{control}-\;\mathrm{GO}\;\mathrm{or}\;\mathrm{MGO}\;\mathrm{sample})\;}{\mathrm{GO}\;\mathrm{or}\;\mathrm{MGO}\;\mathrm{control}}\right]\times \;100$$

The control is composed of MGO or GO without the extract, while the sample is composed of MGO or GO incubated with the extract.

### 2.10. Evaluation of the Extracts’ Capacity to Inhibit AGE Formation

The anti-glycative properties of optimized *A. mollis* extract (before and after chlorophyll removal) were evaluated in the middle step of the glycation reaction. The formation of fluorescent AGEs was monitored by incubating a model system composed of bovine serum albumin (BSA, model protein) and MGO (glycative agent) for 1, 4, and 7 days at 37 °C, in the presence or absence of the extract solution prepared as described in Section 2.9 [20,28]. Aminoguanidine (AG, 0.5 mg/mL) was used as a positive control. The fluorescence intensity (FI) of Argpyrimidine-like (λ_exc_ 335 nm; λ_em_ 440 nm) and Vesperlysine-like (λ_exc_ 370 nm; λ_em_ 440 nm) AGEs was measured, and the ability of the optimized *A. mollis* extract to reduce their formation was calculated as follows:

$$\mathrm{Inhibition}\;(\%)\;=\;\left[1-\left(\frac{\left(\mathrm{FI}\;\mathrm{glycated}\;\mathrm{system}\;(\mathrm{BSA}\;+\;\mathrm{MGO}\right)-\mathrm{FI}\;\mathrm{background}\;(\mathrm{BSA}\;+\;\mathrm{extract}\;)\;}{\left(\mathrm{FI}\;\mathrm{glycated}\;\mathrm{system}\;(\mathrm{BSA}\;+\mathrm{MGO}\;+\;\mathrm{extract}\right)-\mathrm{FI}\;\mathrm{background}\;(\mathrm{BSA})}\right)\right]\times \;100$$

### 2.11. Statistical Analysis

Bioactivity data are reported as mean ± standard deviation (SD) from three independent experiments. The DoE plan was developed using Statgraphics Centurion 19 software (Statgraphics Technologies, Inc., The Plains, VA, USA).

## 3. Results

### 3.1. Identification of the Main Metabolites and Set Up of the UHPLC-UV/DAD Method

Leaves of *A. mollis* were harvested, dried to constant weight and used to prepare a pilot extract. To this aim, a MAE protocol previously developed in-house was applied, setting the parameters as follows: ethanol as a solvent, 50 °C, three cycles of 5 min each.

The dried crude was used to set up a proper (semi)-preparative HPLC-UV/DAD method for the isolation of the main metabolites present in the extract. Particularly, a Chromolith SemiPrep RP-18 endcapped column was selected as the stationary phase, while a mixture of water and methanol in gradient elution was used as the mobile phase. The developed method allowed the isolation of the two main peaks detected in the phytocomplex (retention time 6.60 min and 9.60 min, respectively) in amount and purity suitable for the following identification. Particularly, the ESI-mass spectra of the two analytes suggested that the compound eluting at 9.60 min was compatible with verbascoside. Structure was also confirmed by performing ^1^H NMR analysis and comparing chromatographic and spectroscopic results with a purchased standard. On the other hand, the mass spectrum of the compound eluting at 6.60 min suggests its identification as 2-(2,4-Dihydroxy-1,4(2H)-benzoxazin-3(4H)-one)-β-D-glucoside (DIBOA-glc). Its structure was confirmed by comparing its ^1^H NMR spectrum with the one found in the literature and also by 2D NMR analysis as NOESY. Taken together, all the results obtained were consistent with the identification of the compound eluting at 6.60 min as DIBOA-glc.

**Figure 2:**
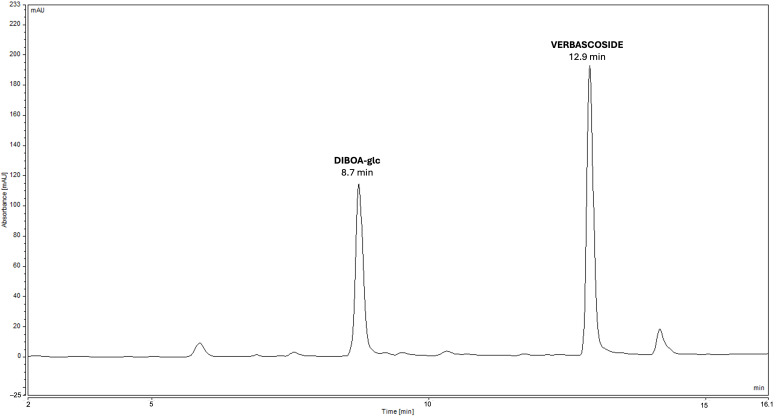
RP-UHPLC-DAD chromatographic profile of *Acanthus mollis* extract recorded at 280 nm.

Once a pilot crude extract and the main metabolites as pure compound were obtained, a proper RP-UHPLC-DAD protocol was set up for the analysis of the phytocomplex and the quantification of the analytes. In particular, the developed RP-UHPLC-DAD method led to an effective resolution and separation of the main metabolites present in the *A. mollis* crude extract, which showed a well-resolved profile, with DIBOA-glc eluting as the first peak (Rt 8.7 min) followed by verbascoside (Rt 12.9 min) (Figure 2). The UV spectra and standard compounds analysis further supported peak identification, as DIBOA-glc showed a characteristic absorption at 257 nm with a shoulder around 280 nm, typical of benzoxazinoid derivatives, while verbascoside exhibited a λmax at approximately 330 nm with a shoulder between 280 and 300 nm, consistent with phenylethanoid glycosides [29]. Method validation was performed according to ICH Q2(R2) guidelines, using the isolated verbascoside and DIBOA-glc. Calibration curves were constructed in the range 1–150 µg/mL and 5–150 µg/mL for verbascoside and DIBOA-glc, respectively, and showed good linearity, with correlation coefficients (R^2^) of 0.9994 and 0.9993, respectively. LOD and LOQ values (0.116 µg/mL and 0.385 µg/mL for verbascoside and 0.113 µg/mL and 0.38 µg/mL for DIBOA-glc, respectively) confirmed good sensitivity for the method. Precision was within acceptable limits, with intra- and inter-day variability ranging from 0.04% to 3.85% for DIBOA-glc, and from 0.03% to 9.16% for verbascoside. Accuracy tests yielded mean recovery values ranging from 99.43% to 101.31% for DIBOA-glc, and from 99.96% to 100.45% for verbascoside. Overall, the obtained data supported the high reproducibility and suitability of the method for the analysis and quantification of *A. mollis* extract metabolites.

### 3.2. Extraction Conditions Optimization

To reach our aim of developing an extract with high verbascoside and DIBOA-glc content from *A. mollis*, a full factorial design was applied by varying the following factors: solvent-to-solid ratio (X_1_), temperature (X_2_), number of extraction cycles (X_3_), and solvent composition (X_4_). The concentrations of the two metabolites in the extracts (computed by means of the calibration curve described in the previous paragraph) were considered as responses: Y_1_ total verbascoside and Y_2_ DIBOA-glc content.

The experimental plan was applied to two different sets of experiments, one applying MAE and the other UAE. Results are summarized in Table 2.

**Table 2:** DoE results. Verbascoside and DIBOA-glc amounts are expressed as mg of metabolite per g of dry weight of the matrix (mg/g DW). Results are expressed as a mean ± SD of three independent injections in UHPLC system.

#	MAE		UAE	
	Verbascoside	DIBOA-glc	Verbascoside	DIBOA-glc
1	190.46 ± 0.14	188.62 ± 4.09	136.32 ± 0.18	198.00 ± 0.38
2	3.63 ± 0.04	162.79 ± 2.84	-	145.89 ± 0.35
3	261.29 ± 0.44	205.18 ± 0.04	29.79 ± 0.02	166.00 ± 0.12
4	184.05 ± 1.38	158.73 ± 0.15	25.82 ± 0.00	142.42 ± 0.01
5	134.47 ± 0.39	150.48 ± 0.72	24.66 ± 0.07	138.71 ± 0.29
6	94.11 ± 1.02	144.80 ± 0.60	62.23 ± 0.21	144.74 ± 0.22
7	216.70 ± 0.78	196.60 ± 4.65	129.88 ± 0.18	205.25 ± 0.24
8	137.93 ± 0.06	160.56 ± 0.10	5.89 ± 0.00	152.12 ± 0.73
9	94.01 ± 0.10	168.22 ± 0.29	-	167.13 ± 0.06
10	170.75 ± 0.36	172.34 ±0.01	100.58 ± 0.11	173.71 ± 2.93
11	163.58 ± 0.54	174.63 ± 0.00	85.56 ± 0.01	164.30 ± 0.13
12	78.19 ± 0.02	174.80 ± 0.12	71.51 ± 0.36	161.06 ± 2.68
13	163.96 ± 0.18	226.85 ± 0.26	99.15 ± 0.57	184.26 ± 1.00
14	84.99 ± 0.03	166.68 ± 0.08	-	148.79 ± 0.26
15	132.88 ± 0.03	177.93 ± 0.12	99.68 ± 0.16	169.87 ± 0.12
16	82.08 ± 0.06	154.13 ± 1.14	20.60 ± 0.03	120.25 ± 1.72
17	114.87 ± 0.24	180.04 ± 0.17	24.83 ± 0.07	140.23 ± 2.63
18	111.60 ± 0.20	168.46 ± 0.08	26.06 ± 0.01	136.57 ± 0.11
19	120.97 ± 0.04	186.49 ± 0.05	31.94 ± 0.06	162.70 ± 0.34

The highest yields of the two metabolites were identified by analyzing the coefficient plots.

Verbascoside and DIBOA-glc recovery (Y_1_ and Y_2_) using either MAE or UAE were significant only considering solvent composition (X_4_), while they were robust across the other tested factors. Furthermore, the MAE approach gave better results when compared with UAE. These results were successfully validated by performing three experiments in the central points of the experimental domain (matrix-to-solvent ratio of 20 mL/mg, 2 cycles, 60 °C, and 50% ethanol in water as solvent). 

### 3.3. UHPLC Analysis of Acanthus mollis Extracts and Its Main Metabolites

Consistently, the optimal extraction conditions were defined as matrix-to-solvent ratio of 10 mL/mg, ramp time of 2 min, extraction time of 5 min, 1 cycle, 80 °C, and 80% ethanol in water as solvent (run 3, extraction yield 19.3%).

These parameters were scaled up to obtain a sufficient amount of extract for further bioactivity evaluations. In particular, we moved from mg to g scale to achieve comparable results, both in terms of total extraction yield and secondary metabolite recovery.

Before proceeding with the bioactivity evaluation steps, an aliquot of the optimized extract was treated to remove chlorophylls using an SPE methodology developed in-house [25].

### 3.4. Bioactivity Evaluations

#### 3.4.1. Anti-Tyrosinase Activity

**Figure 3:**
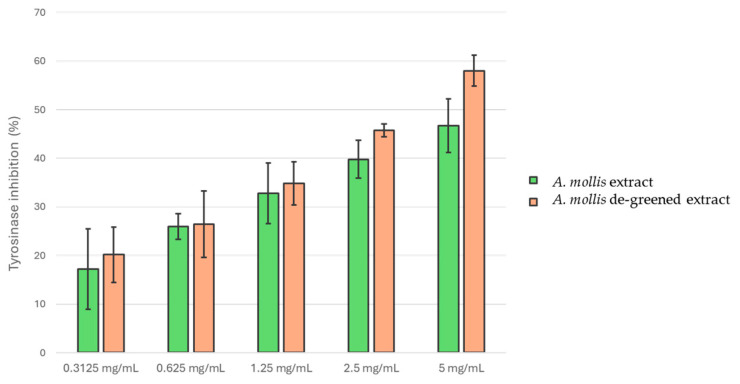
Anti-tyrosinase activity of the extract before and after SPE treatment.

Once the optimized extract and its de-greened aliquot were obtained, a preliminary anti-tyrosinase activity assay was performed (Figure 3). Raw *Acanthus mollis* extract revealed a promising tyrosinase inhibition of approximately 47% ± 11.08%. The chlorophylls removal step enhanced the activity to 58% ± 6.43% (samples tested at 5 mg/mL). Comparison of tyrosinase activity confirmed that the inhibition was statistically significant before and after chlorophyll removal (*t* test, *p* value = 0.0066). This gap between the two values confirms the interfering behavior of chlorophylls in colorimetric assays.

#### 3.4.2. Evaluation of *Acanthus mollis* Extract’s Anti-Glycative Properties

**Figure 4:**
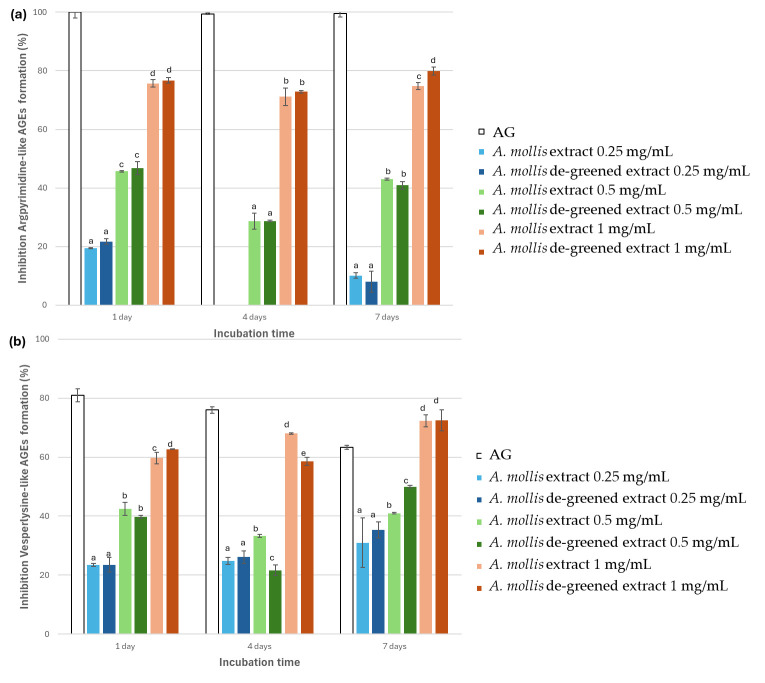
Inhibition (%) of Argpyrimidine-like AGE (**a**) and Vesperlysine-like AGE (**b**) formation in the presence of optimized *Acanthus mollis* extract and de-greened extract at different concentrations (0.25, 0.5, 1 mg/mL). Aminoguanidine (0.5 mg/mL) was used as a positive control. Different superscript letters within each monitoring time indicate significant differences (*p* < 0.05) between extract and de-greened extract.

MGO and GO derive from various metabolic pathways, including the degradation of Amadori products characterizing the middle step of the glycation reaction, glucose autoxidation, and lipid peroxidation, and represent key intermediates in the AGE formation. Due to their high reactivity, these dicarbonyl compounds act as strong glycative agents, reacting primarily with lysine and arginine residues of proteins and thus promoting the formation of AGEs, which are involved in the development of various chronic-degenerative diseases [30,31]. In this context, the anti-glycative properties of the optimized *A. mollis* extract were investigated before and after removing chlorophyll (de-greened extract) to evaluate its capacity to reduce the glycation process in the presence of reactive dicarbonyl compounds. To this end, an in vitro model system composed of BSA (model protein) and MGO (glycative agent) was incubated with the optimized and de-greened extracts tested at different concentrations (0.25, 0.5, 1 mg/mL) to monitor the formation of fluorescent Argpyrimidine-like and Vesperlysine-like AGEs over time (1, 4, and 7 days), according to MGO reaction kinetics [28]. In parallel, the ability of the extracts (0.25, 0.5, 1 mg/mL) to trap dicarbonyl compounds was evaluated by monitoring the levels of the trapped MGO and GO after 24, 48, 72, and 96 h of incubation. As shown in Figure 4a, no significant differences in anti-glycative properties were observed before and after removing chlorophyll (*p* < 0.05), suggesting that the presence of chlorophyll did not affect the extract’s capacity to inhibit Argpyrimidine-like AGE formation. The optimized extract efficiently reduced Argpyrimidine-like AGE formation following a clear dose–response relationship (from 20 to 75%), reaching its highest activity at 1 mg/mL, even if its activity value was lower than that registered for the positive control AG, which completely inhibited Argpyrimidine-like AGE formation at all incubation times. After 4 days of incubation, a slight activity decrease was registered (0% at 0.25 mg/mL; approximately 30% and 70% at 0.5 and 1 mg/mL, respectively), which could be attributed to the several reversible chemical reactions occurring simultaneously during the glycation reaction, which led to a non-linear trend over the monitoring time [32]. Conversely, after 7 days, the inhibitory activity increased again. Overall, the extract’s anti-glycative activity remained nearly constant over time, mainly at the 1 mg/mL concentration. Similarly, the optimized *A. mollis* extract inhibited the Vesperlysine-like AGE formation in a dose-dependent manner, also showing an increase in activity with the increasing of incubation time. In fact, the kinetic profile was opposite to that observed for AG, whose inhibitory activity decreased over time (from 80.98 ± 2.2% to 63.32 ± 0.72%) (Figure 4b). In particular, the extracts tested at 0.25, 0.5, and 1 mg/mL reduced the Vesperlysine-like AGE formation by approximately 20%, 40%, and 60%, respectively, after 1 day. This inhibitory effect was preserved even after 4 days of incubation, in contrast to that observed for Argpyrimidine-like AGEs. After 7 days, the inhibitory activity of 1 mg/mL extract ranged from approximately 30% to 70%, exceeding that of AG. No significant differences were observed between the extract before and after removing chlorophyll, confirming that the presence of chlorophyll did not affect the extract’s anti-glycative properties.

**Figure 5:**
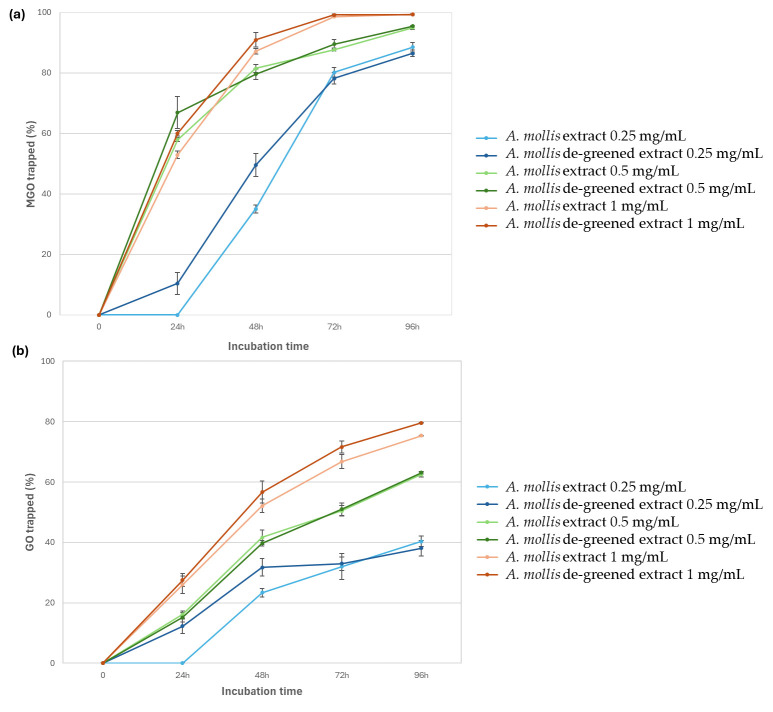
Percentage of MGO (**a**) and GO (**b**) trapped by optimized *Acanthus mollis* extract and de-greened extract at different concentrations (0.25, 0.5, 1 mg/mL).

Concerning trapping capacity, the optimized extract efficiently trapped both MGO and GO, following a clear dose–response relationship (Figure 5). In particular, the trapped MGO increased with incubation time at all tested concentrations, reaching values ranging from 87 to almost 100% after 96 h of incubation, depending on the concentration. Conversely, in the presence of GO, the reaction kinetics appeared slower, with a more gradual increase in the amount of trapped GO over time, reaching values between 40% and 80% after 96 h (Figure 5b). Overall, the extract had high trapping capacity for both MGO and GO, although a higher and faster activity was evident towards MGO, for which high trapping percentages were reached even at lower concentrations and at shorter incubation times. In addition, the extract and the corresponding de-greened sample had an overlapping kinetic profile and comparable trapping efficiency, suggesting that chlorophyll did not affect the trapping capacity.

## 4. Discussion

*Acanthus mollis* L. is a perennial plant belonging to the *Acanthaceae* family. Despite its placement in the BelFrIt list, this plant has not yet been investigated as a potential source of food ingredients. Before investigating the potential bioactivities of its leaf extract, the characterization of its main components was performed. Starting from a pilot extract, a UHPLC method suitable to analyze the phytocomplex from *A. mollis* was developed, highlighting the presence of two main peaks. These peaks were isolated via (semi)-preparative HPLC and characterized as verbascoside and DIBOA-glc. The pure metabolites so obtained were used to further validate the previously developed UHPLC method under both a qualitative and a quantitative point of view. The chromatographic profile obtained is in good agreement with those previously reported in literature for *A. mollis* extracts. In addition, the developed UHPLC method had good selectivity, allowing a clear separation of the two metabolites [16,29].

Based on the already known activities associated with verbascoside and DIBOA-glc, maximizing their concentration in an extract would allow for enhancing the activity against neurodegeneration and aging-related pathologies. To develop an *A. mollis* extract with high verbascoside and DIBOA-glc content, a design of experiment (DoE) approach was adopted, and particularly a full factorial design (number of experiments = 2k, with k being the number of factors) was chosen. The experimental responses were set as the concentrations of the two metabolites in the extract, while the factors were percentage of ethanol as extracting solvent, solvent-to-solid ratio, temperature, and number of extraction cycles. Since the optimized extract is intended for use as a food supplement, different percentages of ethanol were tested as the extracting solvent. Moreover, the solvent-to-solid ratio and the number of extraction cycles were selected as factors in order to maximize the responses but minimize the environmental impact, which are both parameters linked to the amount of solvent required. Also, temperature must be taken into consideration as elevated temperatures reduce the viscosity and surface tension of the solvent, enhancing its capacity to permeate the matrix and improve the solubility of the compounds, but may also cause the degradation of the sample.

Finally, two different extraction techniques were compared: MAE and UAE. Both techniques are considered innovative, efficient, and sustainable, but work with orthogonal methods. While MAE principles are dipolar rotation and ionic conduction, UAE is particularly efficient owing to the cavitation process.

Results of the DoE plan highlighted that MAE is more effective than UAE in extracting higher amounts of verbascoside and DIBOA-glc from the leaves of *A. mollis*. Among the factors considered, the percentage of ethanol as the extracting solvent was the only one that significantly influenced the response. Therefore, extraction conditions were set adopting a MAE protocol with 80% of ethanol in water, 80 °C, one extraction cycle, and the lowest solvent-to-solid ratio. These two last parameters were set to minimize the time and volume of solvent required, thus reducing the environmental impact of the newly developed procedure.

After identifying the optimal conditions, the process was scaled up and an aliquot was treated to remove chlorophylls before bioactivity evaluations by using a methodology developed in-house [25]. The aim is to evaluate if chlorophylls may act as spectrophotometric and fluorescence interfering agents in the anti-tyrosinase and anti-glycative assays, respectively.

Firstly, the optimized *A. mollis* extract and its de-greened aliquot were tested for their anti-tyrosinase activity via a colorimetric enzymatic assay. Results demonstrated that both the extracts inhibited the selected target, even with different potency (raw extract 47%, de-greened extract 58%). Considering that both extracts were tested at the same concentration, these results further demonstrate the interfering behavior of chlorophylls in this assay.

To evaluate the anti-glycative activity of the extract, the reduction in AGE formation was assessed along with the extract’s ability to trap MGO and GO. In particular, the optimized *A. mollis* extract and its de-greened corresponding sample were able to interfere with the glycation process, acting at the middle step of the glycation reaction by reducing AGE formation in presence of MGO, in a concentration-dependent manner, with a progressive increase in activity up to 1 mg/mL, with a comparable activity to that of the positive control AG, a well-known anti-glycative agent. A similar trend was observed in the reduction in MGO and GO levels, with a dose and time-dependent profile indicating the highest tested concentrations (1 mg/mL) efficiently trapping MGO and GO. Given the limited number and range of concentrations tested, these data were used to describe the presence and trend of anti-glycative and carbonyl-trapping activities, but were not considered sufficient to derive reliable IC_50_ values. In contrast to the anti-tyrosinase activity, the de-greened extract exhibited similar activity to that of the untreated one, suggesting that chlorophylls did not act as interfering fluorescence agents in the present assay. The anti-glycative properties of verbascoside are well documented in the literature, largely attributable to its structural components, such as caffeic acid and 3,4-dihydroxyphenylethanol. These compounds can effectively reduce the formation of AGEs, such as carboxymethyllysine and Argpyrimidine, in the presence of several glycative agents (glucose, galactose, and MGO), suggesting a structure–activity relationship [19]. Similarly, plant extracts rich in verbascoside, such as *Scutellaria alpina, Scutellaria altissima*, and *Olea europaea* L., demonstrated a significant capacity to reduce BSA glycation [33,34], as well as *Aloysia citrodora* extract, whose main secondary metabolite is verbascoside, which was able to significantly reduce the AGE formation in the presence of MGO, reaching inhibition levels of up to 60%. This effect is accompanied by a high MGO trapping capacity (up to 100%), suggesting that the reactive dicarbonyls trapping could represent one of the main mechanisms of action involved in the inhibition of AGE formation [20]. These findings were consistent with the results obtained in the present study, as the optimized *A. mollis* extract also had a marked ability to completely trap MGO and almost completely trap GO at the highest concentrations, along with a high capacity to reduce AGE formation. In addition, previous modeling studies have shown that verbascoside was able to recognize and bind the RAGE V domain, forming stable complexes, and further investigation can clarify its effect on RAGE activation, including oxidative and inflammation pathways [12]. Therefore, verbascoside represents a promising anti-glycative agent. However, it shows limited stability under simulated gastrointestinal conditions, which may negatively affect its oral bioavailability, as intestinal digestion promotes its hydrolysis and reduces the bioaccessible fraction [12]. This further highlights the need for an oral formulation able to protect verbascoside during gastrointestinal transit and increase the fraction available for intestinal absorption and systemic distribution. Notably, the bioaccessible fraction of verbascoside displays promising permeability across intestinal epithelial models (Caco-2 cells), without affecting cell viability at concentrations below 600 μg/mL [35,36]. In addition, verbascoside has shown the potential to cross the blood–brain barrier, particularly when formulated to enhance its stability and BBB permeability [37]. Conversely, no previous studies have reported the anti-glycative effects of DIBOA-glc. Its presence in the extract may play a role in the anti-glycative activity, contributing to the overall effect of the extract, as synergistic effects deriving from the combination of different secondary metabolites are often reported [38]. In addition, dietary benzoxazinoids, including DIBOA-glc, showed high bioavailability after oral intake in both animal models and humans, being absorbed through the gastrointestinal tract, detected in plasma, and capable in reaching systemic circulation, as reported in the literature. However, their ability to cross the blood–brain barrier still requires further investigation [39,40].

These results highlight the success of the new protocol in selectively enriching the *A. mollis* extract with key neuroprotective compounds, greatly enhancing its potential application as a future promising ingredient for the development of a neuroprotective food supplement.

## 5. Conclusions

The present study investigates the potential of *Acanthus mollis* as a source of food ingredients potentially able to counteract aging-related neurodegenerative diseases. To this aim, leaves were extracted by applying parameters optimized by the DoE, and the extract was tested as an anti-tyrosinase and anti-glycative agent. Results demonstrated the extract was able to inhibit tyrosinase and to simultaneously reduce the key intermediate-stage glycation markers, specifically MGO and GO levels, alongside inhibit AGE formation. This effect clearly indicates that *Acanthus mollis* optimized extract interferes effectively with the non-enzymatic glycation process, suggesting that its active constituents not only work as carbonyl scavengers but also act upstream to hinder the middle steps of the Maillard reaction.

Despite data from the literature and the presence of the plant in the BelFrIt list that demonstrate the safety of *Acanthus mollis* leaf extracts, deeper investigation will be carried out in future work on the optimization of a valuable oral formulation.

The dual-action mechanism of the optimized *Acanthus mollis* extract, combining anti-tyrosinase activity with potent multi-stage anti-glycative properties, unequivocally confirms its robust bioactivity. In view of these properties, we aimed to investigate the ability of the extract to inhibit tyrosinase activity and suppress induced glycation by using specific cellular models and performing in vivo experiments. This synergistic efficacy establishes *Acanthus mollis* as an interesting starting biomass for future development of food ingredients capable in counteracting aging-related neurodegenerative diseases.

## Figures and Tables

**Table 1:** Experimental plan of the DoE protocol.

#	MAE/UAE Settings				Experimental Matrix			
	Solvent/Solid (mL/g)	T (°C)	Cycles (N°)	Solvent (%EtOH)	Solvent/Solid (mL/g)	T (°C)	Cycles (N°)	Solvent (%EtOH)
1	30	40	3	80	+1	−1	+1	+1
2	30	40	1	20	+1	−1	−1	−1
3	10	80	1	80	−1	+1	−1	+1
4	10	80	3	80	−1	+1	+1	+1
5	10	80	1	20	−1	+1	−1	−1
6	30	80	3	20	+1	+1	+1	−1
7	30	40	1	80	+1	−1	−1	+1
8	10	40	1	20	−1	−1	−1	−1
9	30	40	3	20	+1	−1	+1	−1
10	30	80	1	80	+1	+1	−1	+1
11	10	40	3	80	−1	−1	+1	+1
12	30	80	1	20	+1	+1	−1	−1
13	10	40	1	80	−1	−1	−1	+1
14	10	40	3	20	−1	−1	+1	−1
15	30	80	3	80	+1	+1	+1	+1
16	10	80	3	20	−1	+1	+1	−1
17	20	60	2	50	0	0	0	0
18	20	60	2	50	0	0	0	0
19	20	60	2	50	0	0	0	0

## Data Availability

The original contributions presented in this study are included in the article. Further inquiries can be directed to the corresponding author.

## Abbreviations

The following abbreviations are used in this manuscript:

5-MQ	5-Methylquinoxaline
AG	Aminoguanidine hydrochloride
AGE	Advanced glycation end product
BSA	Bovine serum albumin
DAD	Diode array detector
DIBOA	2,4-dihydroxy-1,4-benzoxazin-3-one
DoE	Design of experiment
ESI	Electrospray ionization
FI	Fluorescence intensity
GO	Glyoxal
HPLC	High Performance Liquid Chromatography
ICH	International Conference on Harmonization
LOD	Limit of detection
LOQ	Limit of quantification
MAE	Microwave-assisted extraction
MAO	Monoamine oxidase A
MGO	Methylglyoxal
OPD	o-Phenylenediamine
RAGE	Receptor for advanced glycation end product
SPE	Solid phase extraction
UAE	Ultrasound-Assisted Extraction
UV	Ultraviolet
